# Spatial Clustering of All-Cause and HIV-Related Mortality in a Rural South African Population (2000–2006)

**DOI:** 10.1371/journal.pone.0069279

**Published:** 2013-07-23

**Authors:** Elias Namosha, Benn Sartorius, Frank Tanser

**Affiliations:** 1 School of Public Health, Faculty of Health Sciences, University of the Witwatersrand, Johannesburg, South Africa; 2 Papua New Guinea Institute of Medical Research, Papua, New Guinea; 3 The Africa Centre for Health and Population Studies, Mtubatuba, South Africa; Vanderbilt University, United States of America

## Abstract

**Background:**

Sub-Saharan Africa bears a disproportionate burden of HIV infection. Knowledge of the spatial distribution of HIV outcomes is vital so that appropriate public health interventions can be directed at locations most in need. In this regard, spatial clustering analysis of HIV-related mortality events has not been performed in a rural sub-Saharan African setting.

**Methodology and Results:**

Kulldorff’s spatial scan statistic was used to identify HIV-related and all-cause mortality clusters (*p*<0.05) in a population-based demographic surveillance survey in rural KwaZulu Natal, South Africa (2000–2006). The analysis was split pre (2000–2003) and post (2004–2006) rollout of antiretroviral therapy, respectively. Between 2000–2006 a total of 86,175 resident individuals ≥15 years of age were under surveillance and 5,875 deaths were recorded (of which 2,938 were HIV-related) over 343,060 person-years of observation (crude all-cause mortality rate 17.1/1000). During both time periods a cluster of high HIV-related (RR = 1.46/1.51, p = 0.001) and high all-cause mortality (RR = 1.35/1.38, *p* = 0.001) was identified in peri-urban communities near the National Road. A consistent low-risk cluster was detected in the urban township in both time periods (RR = 0.60/0.39, *p* = 0.003/0.005) and in the first time period (2000–2003) a large cluster of low HIV-related and all-cause mortality in a remote rural area was identified.

**Conclusions:**

HIV-related and all-cause mortality exhibit strong spatial clustering tendencies in this population. Highest HIV-related mortality and all-cause mortality occurred in the peri-urban communities along the National Road and was lowest in the urban township and remote rural communities. The geography of HIV-related mortality corresponded closely to the geography of HIV prevalence, with the notable exception of the urban township where high HIV-related mortality would have been expected on the basis of the high HIV prevalence. Our results suggest that HIV treatment and care programmes should be strengthened in easy-to-reach high density, peri-urban populations near National Roads where both HIV-related and all-cause mortality are highest.

## Introduction

Identification of areas with excess health problems is important so that appropriate public health interventions can be directed at these locations [1], [2]. Since many diseases are related to location, geographic information systems (GIS) through spatial analysis can identify problem areas or locations for public health effort especially in poor, remote or rural settings which have limited available resources. Evidence suggests that geographic approaches to control and prevention may enhance public health efforts [3] and improve public health care delivery systems. The development of technologies, such as GIS, and the advancement of spatial statistics have allowed the application of not only disease mapping but also spatial analyses, such as spatial clustering, in epidemiological research [4]–[7]. In this context, clusters are defined as a statistically significant excess or deficit of events relative to expectation [8].

There are a large number of studies that have used spatial clustering methods to identify clusters of deaths due to non-communicable diseases such as cancer in developed country contexts, for example, Rosenberg et al [4], and Jemal et al [7]. There are also examples of work that have used spatial clustering methods to help understand the epidemiology of infectious diseases in rural African settings. For example, in our study population, we previously demonstrated substantial geographical heterogeneity in the prevalence of HIV infection [9]. The highest prevalence occurred in peri-urban communities located near the National Road. Snow et al [10], report a space-time clustering of severe childhood morbidity on the Kenyan coast with seasonal peaks in incidence of severe malaria comprising discrete mini-epidemics. Similar studies on micro-epidemiology of malaria were done in Nouna, rural Burkina Faso [1] and the results are likely to help better understand the observed clustering of mortality in the area. However, for deaths related to infection with HIV, spatial clustering has not previously been done at a truly local level.

Here we use population-based data from KwaZulu-Natal, South Africa to detect statistically significant all-cause and HIV-related mortality clusters in a typical rural South African population between 2000 and 2006. In this setting, HIV with or without TB remains the major cause of death among the population [11] and residents are at a high-risk of HIV infection.

## Methods

### Study Area and Population

**Figure 1:**
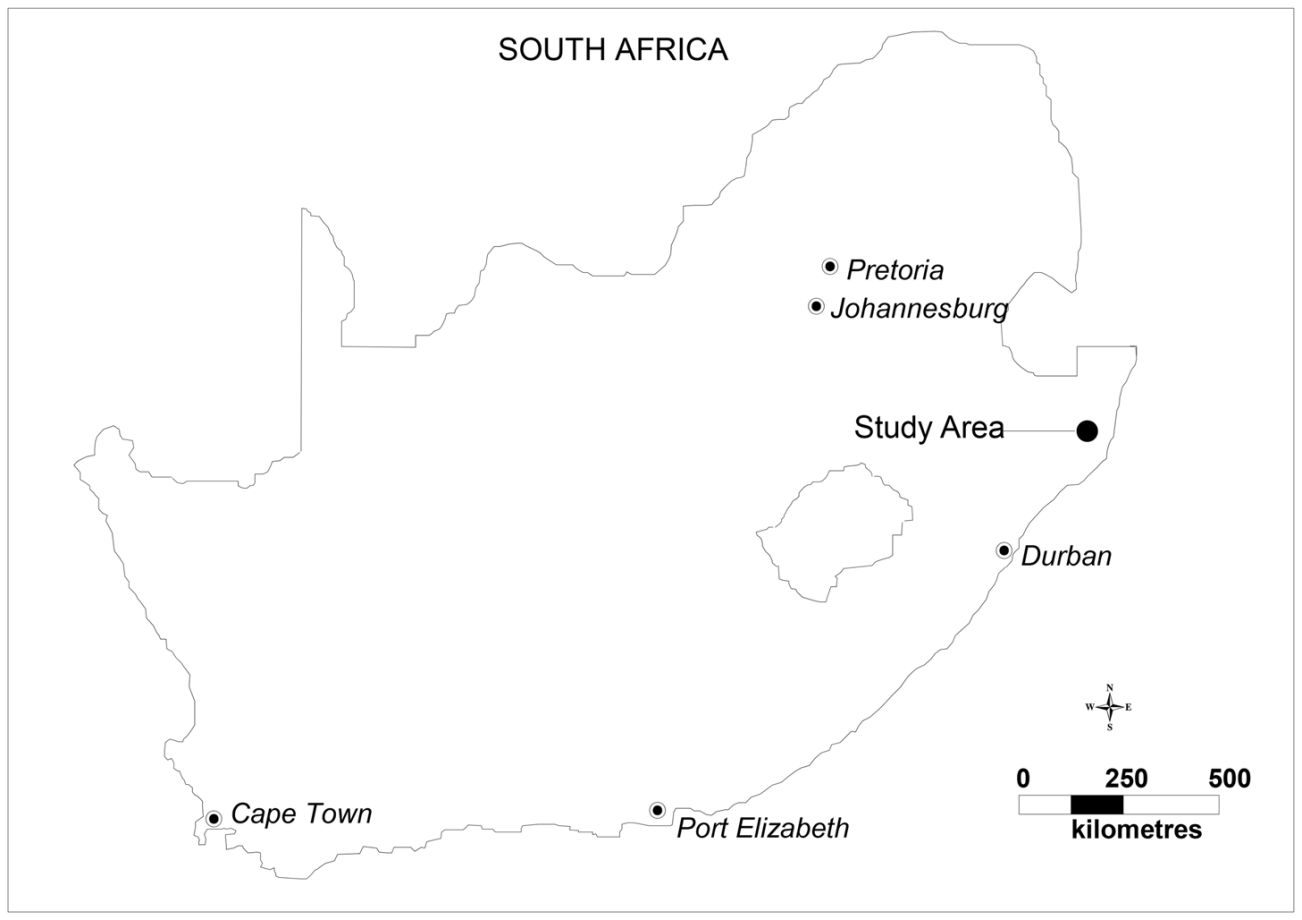
Location of the study area in South Africa.

**Figure 2:**
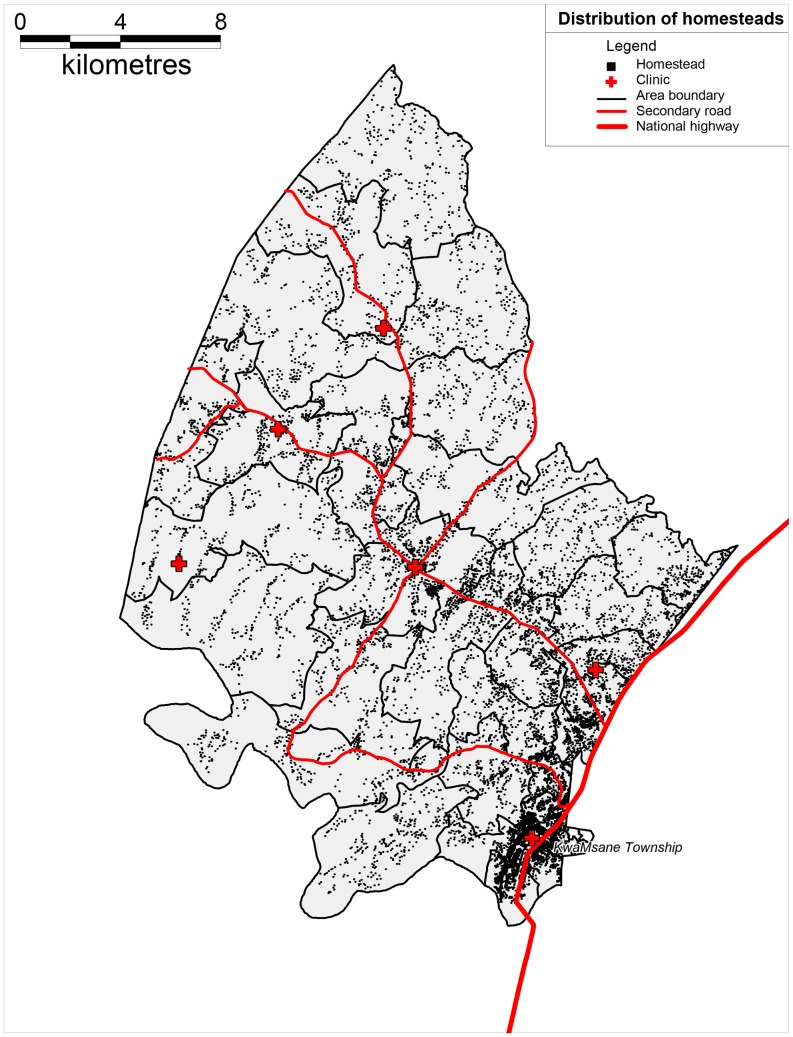
Distribution of homesteads across the study area.

The study area is located near the market town of Mtubatuba in the Umkanyakude district of KwaZulu-Natal province, South Africa (Figure 1) and has been described in detail previously [12]. The population is almost exclusively Zulu-speaking and the study area was part of a former “homeland” under apartheid and is still characterized by high levels of circulatory migration [13]. The adult unemployment rate is 67% and the district of Umkhanyakude has the lowest score of any district in KwaZulu-Natal on United Nation’s Human Development Index [14]. The demographic surveillance site (DSS) was established in 2000 and since then has followed vital events (including all deaths) in a complete population of 87,000. The surveillance area is 438 km^2^, and is typical of many rural areas of South Africa in that while predominantly rural, it contains an urban township and informal peri-urban settlements [12]. All homesteads (N ≈ 12,000) within the Africa Centre’s demographic surveillance area have been mapped via global positioning system (GPS) technology to an accuracy of <2 m (Figure 2). The area is characterized by high HIV prevalence and HIV incidence [15]. Starting in 2004 there has been a rapid roll-out of anti-retroviral therapy (ART), [15], [16] through a nurse-led, devolved public-sector programme described previously [17].

### The Africa Centre Demographic Information System (ACDIS)

ACDIS has been described in detail elsewhere [12]. Briefly, the ACDIS database stores longitudinal health and socio-demographic data on registered subjects, physical structures (e.g. homesteads, clinics and schools) and households [12]. The information is updated every six months through fieldworker visits. The events that are recorded include: individual events (death, birth, migration, etc) household events (household formation, migration, change of household head), and events affecting individual homesteads (start of a new building, change of a building’s main purpose or its owner).

All deaths across the surveillance area are subject to a verbal autopsy interview. The detailed description of this methodology has been described and validated previously [18]. In brief, trained nurses conduct interviews with the caregivers of the deceased. The interview includes an open disease history, a checklist of signs and symptoms, and a structured questionnaire [18]. Two most experienced clinicians then independently assign the cause of death. HIV and TB were combined as a cause of death as it is often difficult to distinguish between them using a verbal autopsy and this improves the sensitivity of the tool. The resulting ICD-10 (10th revision of the International Classification of Diseases) codes were grouped into global burden of disease groups I, II and III [19] with the exception of tuberculosis and AIDS diagnoses, which were classified together into a separate group as HIV-related deaths, given the extensive overlap in mortality from HIV infection and tuberculosis [20]. Ethical clearance for the demographic surveillance and collection of verbal autopsies has been obtained from the University of KwaZulu-Natal’s Ethics Committee.

### Spatial Analysis

The mortality event and person-time data was used to describe the all-cause mortality and HIV-related mortality patterns for the adult (≥15 years of age) population. We aggregated mortality events and person-years of observation (PYO) by *fieldworker area* (48 in total across the study area). Individuals contributed to the person-time denominator from 1 January 2000, or from any later date at which they attained the age of 15, or through in-migration until 31 December 2006, and ceased to contribute to the denominator at death or out-migration. For this analysis we only used person-time accrued and deaths observed whilst the individual was resident at a homestead within the surveillance area. We then calculated directly standardized mortality rates for each fieldworker area to eliminate the influence of age and sex composition by standardizing against the ACDIS population for the period 2000–2006. We displayed this information in the form of thematic maps for both all-cause and HIV-related mortality from 2000–2003 (period before introduction of ART and 2004–2006 (period after introduction of ART), respectively.

### Cluster Detection

Similarly, all mortality events and person-years of observation were summed for all individuals ≥15 years of age in each homestead (mapped to an accuracy of <2 m). We then applied Kulldorff’s spatial scan statistic (Poisson model) implemented in SaTScan software version 7.0 to perform the spatial analysis scanning to detect mortality clusters (high or low mortality clusters) across the surveillance area. A spatial scan statistic is a cluster detection test that is able to both detect the location of clusters and evaluate their statistical significance [21]. This was done by gradually scanning a window across time and/or space across the study area, noting the number of observed and expected observations inside the window at each location using a Poisson model [21], [22]. For any given position of the centre, the radius of the circle changes continuously so that it can take any value. For each circle, the spatial scan statistic calculates the likelihood of observing the observed number of cases inside and outside the circle [21], [23]. The circle with the maximum likelihood is the most likely cluster (least likely to have occurred by chance).

The spatial clustering method [24] offers several advantages: it corrects for multiple comparisons, adjusts for the heterogeneous population densities among the different areas in the study, detects and identifies the location of the clusters without prior specification of their suspected location or size thereby overcoming pre-selection bias, and the method allows for adjustment of covariates [22], [25]. The p-value of the statistic is obtained through Monte Carlo hypothesis testing (9999 iterations), where the null hypothesis of no cluster is rejected at an α level of 0.05 exactly if the simulated p-values is ≤0.05 for the most likely cluster [21], [23]. We limited any possible cluster so it would not exceed 50% of the total population at risk but in practice such a constraint is unlikely to have any bearing on the results given the highly localized spatial heterogeneity in HIV prevalence observed in this population [9]. We ran the analysis by two time periods described previously (2000–2003 and 2004–2006) for HIV-related and all-cause adult population scanning for clusters of high or low mortality rates.

## Results

**Figure 3:**
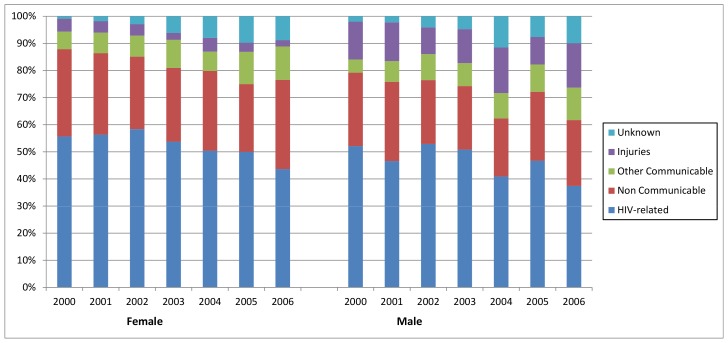
Causes of deaths by year and sex for the adult population (≥15 years of age) between 2000–2006.

Over the seven year period (2000–2006) we observed a total of 86,175 resident individuals ≥15 years of age and 5,875 deaths were recorded (2,938 were HIV-related) over 343,060 PYO. The crude all-cause mortality rate in this population cohort was 17.1 (95% CI 16.7–17.6) deaths per 1000 PYO and was higher in males (18.1 deaths per 1000 PYO) in comparison to females (17.3 deaths per 1000 PYO). Across the two time periods, the crude mortality rate decreased slightly from 17.3 (95% CI 16.7–17.8) deaths per 1000 PYO between 2000–2003 to 17.0 (95% CI: 16.3–17.6) deaths per 1000 PYO between 2004–2006. HIV-related deaths accounted for 45% of deaths in males over 53% for females respectively (Figure 3). Although the HIV-related mortality rate was similar for males and females (≈8.6 per 1000 PYO), HIV-related deaths make up a larger proportion of deaths in females due to the fact that other causes of death such as injury-related deaths are higher in males [18], [26].

**Figure 4:**
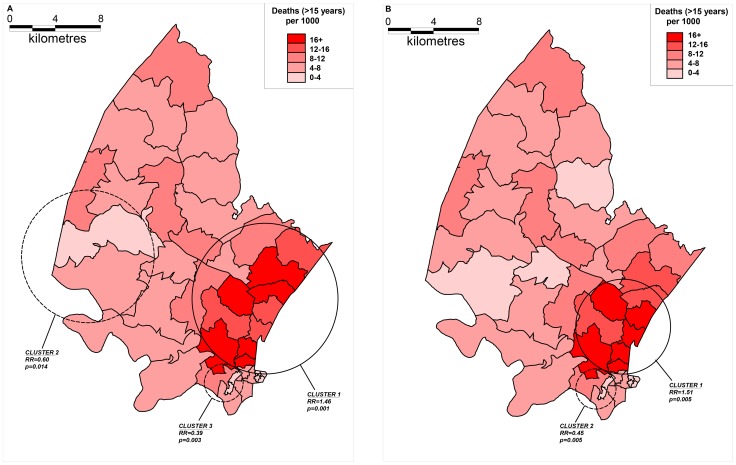
Maps showing HIV-related mortality (≥15 years of age) clusters across the two time periods (A: 2000–2003 and B: 2004–2006) identified by the Kulldorff spatial scan statistic (solid lines indicate high-risk clusters whilst dashed lines indicate low-risk clusters). The clusters are superimposed onto maps showing the corresponding age and sex standardized HIV-related mortality rates aggregated by *“fieldworker area”*.

**Figure 5:**
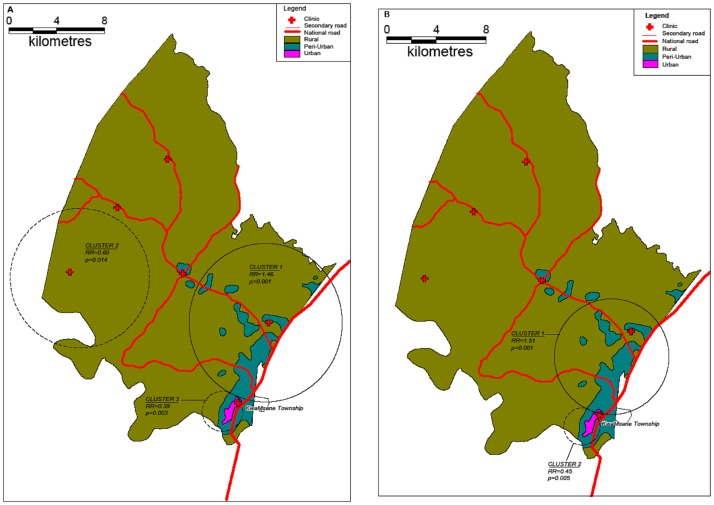
Maps of the study area showing the location of the rural, urban and peri-urban zones. Superimposed on the map are the HIV-related clusters identified during the period 2000–2003 (A) and 2004–2006 (B) identified by the Kulldorff spatial scan statistic. Solid lines indicate high-risk clusters whilst dashed lines indicate low-risk clusters.

The results of cluster analysis for adult HIV-related mortality are shown in Figure 4. All results were adjusted for age and sex. Superimposed on the map of age-standardized HIV-related mortality rates are the clusters identified by the Kulldorff spatial scan statistic for the two time periods (A: 2000–2003 and B: 2004–2006). HIV-related mortality exhibits strong spatial clustering tendencies as measured by the Kulldorff’s spatial scan statistic for time periods 2000–2003 (A, cluster 1, RR = 1.46, *p* = 0.001, 836 observed mortality cases, 673.24 expected) and 2004–2006 (B, cluster 1, RR = 1.51, *p* = 0.001, 323 observed cases, 237.30 expected) respectively. A consistent low-risk cluster was detected around KwaMsane township across both time periods (A, clusters 2 and 3, RR = 0.60, 0.39, *p* = 0.014, 0.003, B, cluster 2, RR = 0.45, *p* = 0.005). The Highest HIV-related mortality occurred in the peri-urban communities along the National Road (Figure 5).

**Figure 6:**
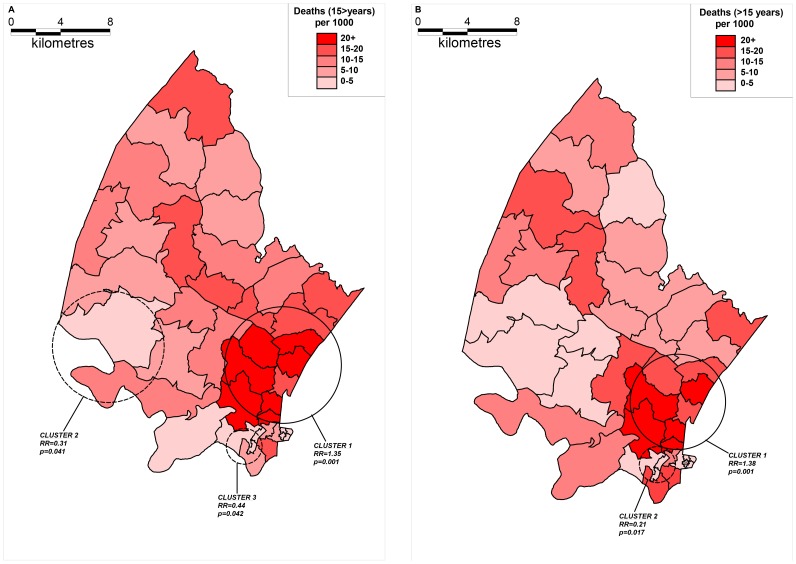
Maps showing all-cause mortality (≥15 years of age) clusters across the two time periods (A: 2000–2003 and B: 2004–2006) identified by the Kulldorff spatial scan statistic (solid lines indicate high-risk clusters whilst dashed lines indicate low-risk clusters). The clusters are superimposed onto maps showing the corresponding age and sex standardized all-cause mortality rates aggregated by *“fieldworker area”*.

The results of cluster analysis for all-cause mortality are shown in Figure 6. Superimposed on the map of age-standardized all-cause mortality rates are the clusters identified by the Kulldorff spatial scan statistic for the two time periods (A: 2000–2003 and B: 2004–2006). Kulldorff’s spatial scan statistic has identified statistically significant clusters with high and low relative risk for both time periods. For time period 2000–2003 (A, cluster 1) a statistically significant cluster was detected with a high RR of 1.35 (*p* = 0.001): 1194 total observed cases (973.66 expected). Low relative risk clusters was also detected (A, clusters 2 and 3, RR = 0.31, 0.44, *p* = 0.041, 0.042). For time period 2004–2006 (B, cluster 1) a statistically significant cluster was detected with a high RR of 1.38 (*p* = 0.001): 660 total observed cases (518.07 expected). A low relative risk cluster was also detected for the period 2004–2006 (cluster 2) with a RR of 0.21, *p* = 0.017.

All-cause mortality clusters were located in similar locations as the HIV-related mortality clusters. This largely reflects the fact that HIV-related mortality is the major cause of death in this population and hence the spatial patterns of all-cause mortality are influenced by the underlying spatial distribution of HIV-related mortality.

## Discussion

We have investigated for the first time localized spatial clustering of HIV-related and all-cause mortality events in adults (≥15 years of age) in a typical rural South African population with high HIV-prevalence. Both HIV-related and all-cause mortality are not homogenously (randomly) distributed in this population and exhibit strong spatial clustering tendencies as measured by the Kulldorff spatial scan statistic. Highest HIV-related and all-cause mortality occurred in the peri-urban communities along the National Road and were lowest in the urban township and remote rural communities. Overall, these results provide a clear rationale for the need to strengthen HIV treatment and care programmes in the high density peri-urban communities along the National Road.

Our results reinforce the substantial contribution of HIV to all-cause mortality in this population with half of all adult deaths over the study period being HIV-related. Our findings are supported by previous research in the same population which showed a reduction in HIV-related mortality post 2004 [26]. The high HIV mortality clusters identified in this study corresponded approximately with areas of high HIV prevalence identified previously in this population in communities closest to the National Road [9]. Indeed, a strong ecological relationship between proximity to roads and HIV prevalence among women attending antenatal clinics has been previously demonstrated in this setting [27]. Interestingly, the low mortality cluster around the urban township (close to the National Road) corresponded to a cluster of relatively high HIV prevalence. This could be a consequence of a combination of better living conditions, [28], [29] and easier access to HIV treatment and care in the urban area in comparison to the surrounding peri-urban communities. The locations of high and low mortality communities were remarkably similar across the two time periods. This reflects the fact that mortality rates in the peri-urban communities were always relatively higher than the surrounding population (and the converse was true for the urban and deep rural communities) despite the fact that overall mortality had decreased slightly across the two time periods. In line with our previous work [9], the results provide further evidence to challenge the paradigm of a ubiquitous ‘generalized’ rural epidemic. Rather the marked spatial variation in mortality in this population is likely a consequence of the fact that several localized HIV sub-epidemics occur in this population that are partly contained within geographically defined communities. Consequently, resources should not be distributed in a purely uniform manner in such settings where clear, marked geographical variations in mortality exist.

The last two decades have seen substantial work in the GIS arena on the statistical analysis of point patterns, e.g, Besag and Newell (1991) [3], and spatial clustering is one of the many statistical analysis used for point pattern analysis. Spatial clustering detection methods are classified as global, local and focused [3], [30]. In this present study we chose the local spatial scan statistic (Kulldorff’s spatial scan statistic) over others [5], [31], as it is suitable for the purpose of this present study because it determines the existence of statistically significant clusters and their geographic locations. Quite often public health authorities need to respond to demands to investigate potential clusters of different diseases and confirm or refute, with certainty, whether a health problem exists in a particular location [23]. The application of GIS and spatial scan statistics can help health workers better understand the HIV-related mortality patterns both in space and time.

A strength of our cluster detection approach is that we do not aggregate the data by arbitrary (with respect to the “boundary” of the cluster) administrative units but instead use person-exposure and mortality events at the level of individual homesteads (accuracy <2 m) to define the "clusters". A limitation of the analysis using the Kulldorff’s spatial scan statistic is that clusters are defined as circles [1], [10]. For example, if a homestead with a lower mortality rate is located next to or is surrounded by homesteads with a higher mortality rates it is more likely that the homestead with a lower mortality rate will always be included in the cluster [32], [33].

The results of this present study can be used to target high-risk communities for public health intervention and prioritize the areas that need thorough epidemiological investigation. Although the detailed datasets used in this analysis are unlikely to be available at a National level, the principal elucidated in our work suggests that HIV treatment programmes should be strengthened in easy-to-reach high density, peri-urban populations near National Roads where mortality rates are highest.
